# Rapid establishment of laboratory diagnostics for the novel coronavirus SARS-CoV-2 in Bavaria, Germany, February 2020

**DOI:** 10.2807/1560-7917.ES.2020.25.9.2000173

**Published:** 2020-03-05

**Authors:** Regina Konrad, Ute Eberle, Alexandra Dangel, Bianca Treis, Anja Berger, Katja Bengs, Volker Fingerle, Bernhard Liebl, Nikolaus Ackermann, Andreas Sing

**Affiliations:** 1 Public Health Microbiology Unit, Bavarian Health and Food Safety Authority, Oberschleißheim, Germany; 2 These authors contributed equally to this work; 3 Unit of Virology, Bavarian Health and Food Safety Authority, Oberschleißheim, Germany; 4 State Institute of Health, Bavarian Health and Food Safety Authority, Oberschleißheim, Germany; 5 Ludwig Maximilians-Universität, Munich, Germany

**Keywords:** SARS-CoV-2, Molecular Testing, Laboratory Diagnostics, Real-Time PCR, COVID-19, Corona Virus, Infectious disease, outbreaks

## Abstract

The need for timely establishment of diagnostic assays arose when Germany was confronted with the first travel-associated outbreak of severe acute respiratory syndrome coronavirus 2 (SARS-CoV-2) in Europe. We describe our laboratory experiences during a large contact tracing investigation, comparing previously published real-time RT-PCR assays in different PCR systems and a commercial kit. We found that assay performance using the same primers and probes with different PCR systems varied and the commercial kit performed well.

At the end of December 2019, an outbreak caused by a novel coronavirus was announced in Wuhan, China. Since then, the number of cases has increased, especially in China but also in other countries, and public health authorities are in need to rapidly implement diagnostic tools. In this paper, we describe our laboratory experiences with the novel real-time RT-PCR assays comparing different one-step PCR systems and a commercial kit, using a Bio-Rad CFX 96 cycler.

## Timely implementation of molecular diagnostics for SARS-CoV-2

In connection with the ongoing outbreak of a novel coronavirus in the province Hubei and surrounding areas in China, it was expected that Europe would also be confronted with the emerging severe acute respiratory syndrome coronavirus 2 (SARS-CoV-2), as infections in travellers in several Asian countries outside of China were confirmed shortly after the announcement of the outbreak in Wuhan [1-4]. Therefore, it was necessary to rapidly implement adequately quick and sensitive diagnostic assays for outbreak management of SARS-CoV-2 in public health laboratories.

As soon as the World Health Organization (WHO) published the first protocols for real-time RT-PCR assays, the Bavarian Food and Health Authority started to implement them. We ordered control material and oligonucleotides (see details below) in week 4 and ran our first SARS-CoV-2 assays on 27 January (week 5). On the same day, the first German case of coronavirus disease 2019 (COVID-19) was diagnosed in Bavaria [1]. In the following days, health authorities implemented comprehensive measures to prevent further transmission of SARS-CoV-2, including testing of contact persons. 

We initially used the protocol based on the E gene and RdRp gene developed by the German Consiliary Laboratory for Coronaviruses hosted at the Charité in Berlin [5]. Real-time RT-PCR was initially performed with the QuantiTect Virus +Rox Vial kit (QIAGEN, Hilden, Germany) on the Bio-Rad CFX96 Touch Real-Time PCR Detection System (Bio-Rad, Feldkirchen, Germany). The kit was chosen for its frequent and successful use in our laboratory with other assays and its immediate availability. Primers and probes were used as described [5] and provided by TIB Molbiol (Berlin, Germany). Control material was ordered from the European Virus Archive (EVAg) and consisted of synthetic Wuhan coronavirus 2019 E gene control (reference number 026N-03866) and SARS-CoV Frankfurt 1 RNA (reference number 004N-02005) [6]. In addition, the control of LightMix Modular Wuhan CoV RdRP-gene (TibMolbiol, Berlin, Germany) was used for the SARS-CoV-2 specific assay. Respiratory samples (nasopharyngeal swabs or sputum) were obtained from patients and contact persons. Sputum samples were diluted in 2 mL phosphate buffered saline (PBS). RNA was extracted using the QIAamp Bio Robot kit (QIAGEN) on a Hamilton Microlab Star (Hamilton, Bonaduz, Switzerland).

Each sample was tested by three PCRs: the screening assay for the E gene and two confirmatory assays targeting the RdRp gene, all performed as recommended in [5], with either both probes (RdRP_SARSr-P1 and RdRP_SARSr-P2), detecting SARS-CoV and SARS-CoV-2, or only with the SARS-CoV-2-specific probe RdRP_SARSr-P2 [5]. We used human RNAse P as control for RNA extraction [7].

**Figure 1:**
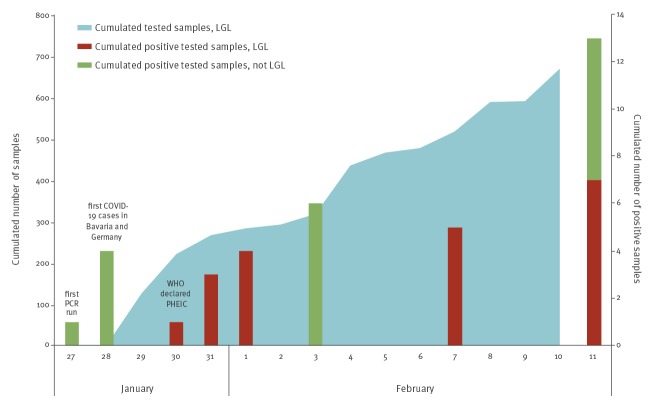
Cumulative numbers of suspected COVID-19 samples tested during week 5 and 6 2020, Bavaria (n = 675)

COVID: coronavirus disease; PHEIC: public health emergency of international concern; LGL: Bavarian Health and Food Safety Authority; WHO: World Health Organization.

The cumulative numbers of all tested samples are shown in Figure 1. Two weeks after starting the SARS-CoV-2 diagnostics, we had analysed 669 respiratory samples at the Bavarian Health and Food Safety Authority (LGL). Among them, seven showed positive results for SARS CoV-2 RNA. By 11 February, 14 COVID-19 cases had been detected in Bavaria.

The figure shows all samples tested at LGL, negative and positive, as well as six positive samples tested in other laboratories in Bavaria. All data are cumulative, with a final total of positive tests of 13.

## Comparison of one-step RT-PCR systems

**Figure 2:**
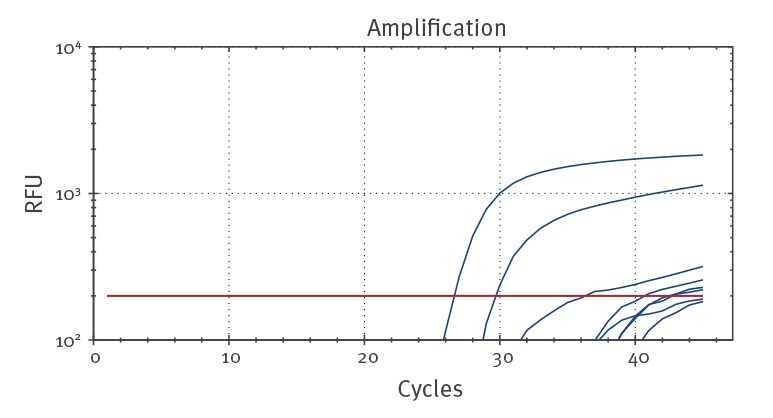
Example image of real-time RT-PCR curves of the E gene assay with unspecific signals at late cycles, Bavaria, February 2020

During the 2 weeks after the first PCR test on 27 January, a rapidly increasing number of case contacts in Bavaria was identified and their respiratory samples were tested. We found that the SARS-CoV E gene screening assay with the QuantiTect Virus +Rox Vial kit showed moderate to high amounts of unspecific signals in late cycles in 61% (451/743) of the tested patient samples and also of negative extraction and non-template controls (Table, Figure 2), which complicated the evaluation of the qPCR result. The RdRp assays were basically free from such unspecific signals in late cycles. Cycle threshold (Ct) values of the control SARS-CoV Frankfurt 1 RNA were reached three cycles earlier in E gene assay than in RdRp assays. 

**Table:** Comparison of two different one-step real-time RT-PCR systems with SARS-CoV-2 assays from Corman et al. [5] and a commercial test kit with kit-specific assays, Bavaria, February 2020

Real-time RT-PCR system	PCR efficiency (%)^a^, linearity (R^2^)	Limit of detection (copies/reaction)	Unspecific signals count in E gene assay in total^b^	Unspecific signals in E gene assay (%)^b^	Run time (hours)
QuantiTect Virus +Rox Vial kit (QIAGEN)	ND	ND	451/743 (75/126 NC, 376/617 patient samples)	60.7	1:50
SuperScript III One-step RT-PCR System with Platinum TaqDNA Polymerase (Invitrogen)	95 / 0,99^c^	50^c^	13/257 (2/38 NC, 11/219 patient samples)	5.1	1:28
RealStar SARS-CoV-2 RT-PCR kit 1.0 (Altona)	125 / 0,97^d^	10^d^	0/111 (0/38 NC, 0/73 patients samples)	0	2:15

NC: negative control samples; ND: not determined.

^a^ E = 10^−1/slope^ − 1.

^b^ Indicated counts and percentage values of unspecific background signals in the SARS-CoV E gene assay are based on the total number of tested patient samples as well as the negative extraction and non-template controls.

^c^ Only for RdRp gene assays, tested with four replicates of SARS-CoV Frankfurt 1 RNA [6]; 10-fold serial dilutions were determined. For the E gene, the assay was not linear.

^d^ Only for the E gene, tested with two replicates of synthetic Wuhan coronavirus 2019 E gene control and SARS-CoV Frankfurt 1 RNA each [6]; 10-fold serial dilutions were determined.

RFU: relative fluorescence units.

Curves: 1: Wuhan coronavirus 2019 E gene positive control; 2: SARS-CoV Frankfurt 1 RNA positive control; 3,4,6,8: negative patient samples; 5: extraction negative control; 7: non-template negative control.

Signal is given in log scale with threshold = 200. PCR was performed with SuperScript III system and E gene primers and probe as published in [5]. Curves of positive controls (1 and 2) show expected sigmoid curves. Curves 3–6 show unspecific signals with increase above threshold. Curves below threshold were not considered as significant signals (7 and 8).

Three additional one-step real-time PCR systems were compared with the initially used QuantiTect Virus +Rox Vial kit (QIAGEN) (Table): (i) OneStep RT-PCR kit (QIAGEN; data not shown in the Table because only a limited number of samples were tested in only one run), (ii) LightCycler Multiplex RNA Virus Master (Roche, Mannheim, Germany; data not shown because only a limited number of samples were tested in only one run) and (iii) SuperScript III One-Step RT-PCR system with Platinum TaqDNA Polymerase (Invitrogen, Darmstadt, Germany) (Table). Each assay protocol and thermoprofile was adjusted to the recommendations of the manufacturer, but primer and probe concentrations were the same as published [5]. They all showed comparable results in reduction of unspecific E gene signals. Using SuperScript III, the unspecific signals in the E gene screening assay were significantly reduced to 5% (13/257) in tested patient samples and negative extraction and non-template controls (Table), whereas Ct values for positive controls (Wuhan coronavirus 2019 E gene and SARS-CoV Frankfurt 1 genomic RNA [3]) were in the same range in QuantiTect (median: 29.1; range: 26.8–32.4; n = 85) and Superscript (median Ct: 28.1; range: 26.4–31.0; n = 30) setups. 

In the following, we decided to switch to the SuperScript III system as recommended by Corman et al. [5], also because of its decreased thermoprofile run-time (Table). Moreover, we additionally included a newly launched commercial test kit in our study: RealStar SARS-CoV-2 RT-PCR kit 1.0 (Altona, Hamburg, Germany), which did not show unspecific E gene signals. A summary of the assay features of the three PCR setups tested and compared in detail is shown in the Table.

For evaluation of assay performance regarding efficiency, linearity and unspecific signals, we used the SuperScript protocol in a direct comparison of 73 samples with the RealStar SARS-CoV-2 assay. The E gene assays showed 97% (71/73) identical results in both assays (60 negative and 11 positive results as well as two divergent results, both with a positive Real Star SARS-CoV-2 assay and a negative SuperScript protocol assay). The specific SARS-CoV-2 assays gave in 67 (92%) identical results (60 negative and seven positive) and six divergent results. Overall, the RealStar SARS-CoV-2 kit seemed to be more sensitive. 

## Workflow

**Figure 3:**
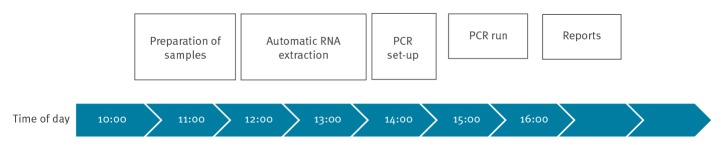
Flowchart and routine timeline of the SARS-CoV-2 diagnostic workflow at the LGL, Bavaria, February 2020

With increasing sample numbers, it is crucial to implement a new assay into routine laboratory workflows that already exist for diagnostic testing of other pathogens. Sample preparation and RNA extraction were carried out at BSL2 level under a safety class 2 cabinet wearing FFP3 filter masks following the WHO recommendations [8]. Samples arriving at our laboratory before 10:00 h were analysed on the same day and results were reported 6–7 h later to local health authorities (Figure 3). Our daily test capacity was ca 80 samples. It should be taken into account that well trained and experienced personnel is needed and seasonal pathogens, e.g. influenza and noroviruses, had to be analysed in parallel. Overall, we managed to report the results for 97% of samples within the same day.

LGL: Bavarian Health and Food Safety Authority.

## Discussion

On 7 January 2020, a novel coronavirus was identified and shortly after, the first sequence of the new strain was published [9,10]. The main task of public health authorities is to react quickly to emerging pathogens of global threat to prevent spread. These responses include containment strategies, which means that close contacts of patients have to be identified and tested immediately. Reusken et al. identified the availability of positive control material and primer/probes as well as the lack of skilled personnel and time as the most prominent challenges in the implementation of the new SARS-CoV-2 assay [11].

National and local public health laboratories play a crucial role in testing emerging pathogens. The Public Health Microbiology Laboratory in Bavaria was confronted with SARS-CoV-2-related events very early: once the assays and control materials arrived and the PCR assays were performed for the first time, a large contact investigation around the first German COVID-19 patient (data not shown) was immediately started, with so far more than 700 samples. Evaluation and verification of the new assays and testing real samples had to take place simultaneously within a very short time. 

We realised soon that the SARS-CoV E gene assay was more sensitive than the two RdRp gene assays combined with the one-step RT-PCR system available in our laboratory. However, our E gene assay showed high background levels hampering a clear evaluation of the assays. Each mastermix has its own proprietary composition, which may explain the differences in the performance of a certain PCR assay. Using commercial kits with optimised target regions and primer sequences (in the E gene and SARS-CoV-2-specific S gene) ruled out the unspecific signals completely. Hence, reasons for the observed unspecific signals may be dimerisation of primers and probes and/or unspecific primer binding and polymerase activity in the targeted region of the E gene, probably also depending on thermal profile and cycler-specific differences, or most likely a combination of these factors.

Contamination as a reason for unspecific signals was ruled out, as stringent prevention measures were taken, e.g. strict separation of working areas: oligonucleotides and PCR mastermix reagents were handled in one room under a PCR hood with specified laboratory coats. Sample preparation and RNA extraction took place in a second room. Sample RNA was added in a third room under a PCR hood. The synthetic E gene control was added last to the mastermix. All reagents were aliquoted and aliquots used once only. Contaminations from synthetic E gene present in primer batches upon delivery can be ruled out as well, although only one batch of E gene primers and probes was used with the QuantiTect and Superscript III setup, as only a certain proportion of samples showed the unspecific signals. Furthermore, the unspecific signals were significantly reduced in the Superscript III setup, which showed that its sensitivity was comparable to the QuantiTect setup. In addition, the initially used E gene primers and probe were separately used as templates with the RealStar kit and no amplification was observed, whereas the corresponding artificial E gene template delivered a clear S-shaped curve with this kit.

## Conclusion

The SARS-CoV-2 assay from Corman et al. [7] could be used rapidly with the published SuperScript III system, but further optimisation especially of the E gene assay may enhance the sensitivity. The RealStar kit outperformed the two other tested one-step PCR systems in sensitivity and by absence of unspecific signals with the used primer set and the RealStar workflow enhanced laboratory efficiency combining two coronavirus assays (lineage B CoV and specific SARS-CoV-2) with an IC in a triplex PCR. Overall, fast assay development and publication of protocols by expert laboratories allowed public health laboratories to establish diagnostics quickly and act fast. Assay optimisation will further speed up and improve the management of this ongoing outbreak.
